# Inspecting the True Identity of Herbal Materials from *Cynanchum* Using ITS2 Barcode

**DOI:** 10.3389/fpls.2017.01945

**Published:** 2017-11-13

**Authors:** Mengyue Guo, Li Ren, Xiaohui Pang

**Affiliations:** Key Laboratory of Bioactive Substances and Resources Utilization of Chinese Herbal Medicine, Ministry of Education, Institute of Medicinal Plant Development, Chinese Academy of Medical Sciences and Peking Union Medical College, Beijing, China

**Keywords:** *Cynanchum*, herbal materials, DNA barcoding, identification, ITS2

## Abstract

*Cynanchum* is a large genus with some important medicinal species in China. The medicinal species in *Cynanchum* are easily confused, leading to potential safety risks. In this study, the internal transcribed spacer 2 (ITS2) barcode was used to discriminate the medicinal plants in *Cynanchum*. The identifying capability of ITS2 was assessed using the specific genetic divergence, BLAST1, neighbor-joining (NJ) tree, maximum-likelihood (ML) tree, and single-nucleotide polymorphism (SNP) methods. Results indicated that the intra-specific genetic divergences of *Cynanchum* species were lower than their inter-specific genetic divergences. Of the 87 samples from 17 species, ITS2 showed a high identification efficiency of 90.8 and 87.4% at the species level through BLAST1 and the nearest distance methods. NJ tree and ML tree also demonstrated the suitability of ITS2 to differentiate *Cynanchum* species. Meanwhile, a stable SNP was found, and it could accurately authenticate *Cynanchum paniculatum* and *Cynanchum atratum*. Furthermore, we collected 64 commercial samples from three commonly used herbal medicines and evaluated the capability of ITS2 to survey their authentication. Of these samples, Cynanchi Atrati Radix et Rhizoma (Baiwei) showed a potential safety problem, and all the 11 test samples were adulterants. In conclusion, ITS2 can distinguish medicinal species in *Cynanchum* effectively, and its application could greatly improve the identification efficiency and accuracy of commercial herbal medicines in this genus.

## Introduction

Traditional Chinese medicine (TCM), an integral part of Chinese culture, plays a predominant role in the healthcare system of China, and it is recognized as a primary treatment strategy. Herbal medicine accounts for more than 80% of Chinese medicine resources, taking an essential part in the TCM system and medicine market. Meanwhile, herbal medicines are used as a complementary and alternative medicine (CAM) worldwide (Zhu and Woerdenbag, 1995; Eisenberg et al., 1998; Ernst and White, 2000; Bensoussan and Lewith, 2004). However, counterfeit drugs, misidentified drugs, and mislabeled drugs are sold in the market prevalently, resulting in side effects and drug resistance. Xin et al. (2015) surveyed commercial *Rhodiola* products and found that only 40% of samples are authentic *Rhodiola crenulata*, indicating potential risks and safety problems of medicine use. Han et al. (2016) investigated 295 medicinal species, including 1,436 samples from seven primary TCM markets in China, and identified that about 4.2% of the samples were adulterants. These studies highlighted the urgency of accurate identification of herbal medicines.

*Cynanchum* of the family Asclepiadaceae is a large genus with about 200 species that are widely distributed in Africa, North America, South America, Asia, and Europe. *Cynanchum* consists of 57 species in China, 19 of which are used as herbal medicines. For centuries, medicinal plants in *Cynanchum* have been applied for the prevention and treatment of various diseases, with C21 steroidal glycosides as the major active components (Gu and Hao, 2016). Cynanchi Paniculati Radix et Rhizoma (Xuchangqing), which originated from the dried roots and rhizomes of *Cynanchum paniculatum*, is used to relieve rheumatic arthralgia, lumbago, and other types of pain and exhibits anti-inflammatory, anti-nociceptive, and neuroprotective activities (Choi et al., 2006; Weon et al., 2012). Cynanchi Stauntonii Rhizoma et Radix (Baiqian), derived from dried rhizomes and roots of *Cynanchum stauntonii* or *Cynanchum glaucescens*, is used for descending Qi, relieving cough, and expelling phlegm. Its anti-inflammatory, antiviral, and antitussive activities have been reported as well (Yang et al., 2005; Yue et al., 2014; Yu and Zhao, 2016). The dried roots and rhizomes of *Cynanchum atratum* and *Cynanchum versicolor*, known as Cynanchi Atrati Radix et Rhizoma (Baiwei), have been used for clearing heat to cool the blood, disinhibiting urine to relieve stranguria, and removing toxin to treat sore. Meanwhile, Baiwei displays anti-inflammatory, cell apoptotic regulating, and acetylcholinesterase inhibitory activities (Lee et al., 2003; Jeon et al., 2011; Zhang et al., 2015). Bunge Auriculate Root (Baishouwu) is an appellative name for the root tubers of *Cynanchum auriculatum, Cynanchum bungei*, and *Cynanchum wilfordii* in China, which is a famous tonic drug in TCM and known for its functions in enriching vital essence and enhancing immunity (Shan et al., 2006). Pharmacological studies suggested that Bunge Auriculate Root exhibits antitumor, antidepressant, anti-inflammatory, and antiepileptic activities (Peng et al., 2008; Yang et al., 2011, 2014; Li et al., 2016). In addition, the root of *Cynanchum otophyllum* (Qingyangshen) has antifungal and antiepileptic activities (Zhao et al., 2007). All the parts of *Cynanchum chinense* (Erongteng) are used to treat colds and chills (Yu et al., 2015). *Cynanchum wallichii* (Duanjieshen) is used in the famous Chinese prescription “Hulisan” as a primary drug to treat arthrophlogosis and injury from fall or fracture (Zhang and Zhou, 1983).

In general, species in the same genus feature similar morphological characteristics. However, the highly similar morphological features of many *Cynanchum* species complicate species discrimination in this genus, resulting in herbal medicine confusion. For example, Cynanchi Paniculati Radix et Rhizoma, Cynanchi Stauntonii Rhizoma et Radix, and Cynanchi Atrati Radix et Rhizoma are all recorded in Chinese pharmacopeia (2015 edition) and are the most commonly used herbal medicines from *Cynanchum*. These medicines are very similar in appearance that they are usually used incorrectly or in confusion. However, these three medicines generate completely different effects, leading to safety problem when they are misused in clinical practice. The herbal medicine Bunge Auriculate Root (Baishouwu) is under the same circumstance. The original plants of Baishouwu are *C. auriculatum, C. bungei*, and *C. wilfordii* in China as mentioned above. In Korea, Baishouwu is registered in Korea Herbal Pharmacopeia as Cynanchi Wilfordii Radix (Baek Su O), used as food and traditional herbal medicine. *C. wilfordii* is the only original plant of Baek Su O, whereas *C. auriculatum* is considered an adulterant (Kim et al., 2005). The cut and dried roots from *C. wilfordii* and *C. auriculatum* in the Korean herbal market are commonly misused because of their similar morphology (Li et al., 2013). Aside from morphology factor, homonym is another cause of herbal medicine confusion. One typical case is that *C. wallichii* and *C. otophyllum* are both sold with the name Qingyangshen in the market. However, their effects differ from each other completely (Zhang and Zhou, 1983; Zhao et al., 2007). Confusion between them is a latent threat to the safety and interests of consumers.

At present, relevant studies for the identification of *Cynanchum* species were limited, and these studies are all focused on the discrimination between *C. auriculatum* and *C. wilfordii* (Moon et al., 2009; Ryuk et al., 2014; Lee et al., 2015). Therefore, establishing an effective method to distinguish the herbal materials from *Cynanchum* systematically is necessary to avoid incorrect prescriptions. DNA barcoding is a convenient, accurate, and rapid tool to identify species by using a short fragment of the genomic sequence. This tool has aroused great concern (Gregory, 2005; Schindel and Miller, 2005; Miller, 2007) since it was first proposed by Canadian zoologist Paul Hebert in 2003 (Hebert et al., 2003). DNA barcoding is a reliable technique to authenticate species on the basis of DNA sequences and is thus not influenced by factors such as the morphological characteristics, plant parts, and age of samples. Hence, this technique allows non-experts to identify an unknown species without professional taxonomic knowledge. Recently, DNA barcoding has been broadly recognized and widely applied in the discrimination of plants. Kress et al. (2005) recommended the nuclear internal transcribed spacer region and the plastid *trnH*-*psbA* intergenic spacer as potential barcodes for flowering plants. Lahaye et al. (2008) analyzed more than 1,600 samples and suggested a portion of the plastid *matK* gene could be a universal DNA barcode for flowering plants. Then, CBOL Plant Working Group (2009) proposed the 2-locus combination of *rbcL* + *matK* as the barcode for land plants. In 2010, Chen et al. (2010) identified more than 6,600 samples obtained from 4,800 species in 753 genera with 92.7% identification rate by using internal transcribed spacer 2 (ITS2) and suggested ITS2 to be a novel barcode for medicinal plants. Subsequently, China Plant BOL Group (2011) assessed the effectiveness and universality of four markers (*rbcL, matK, trnH-psbA*, and ITS) in 1,757 species of 141 genera and proposed that ITS/ITS2 should be incorporated into the core barcode for seed plants. In accordance with the proposal of China Plant BOL Group, nrDNA ITS was suggested as a potential barcode for plant species in Hollingsworth's research as well (Hollingsworth, 2011). Furthermore, Pang et al. (2011) showed that ITS2 is superior in species identification than the three other DNA barcodes (*rbcL, matK*, and *rpoC1*) for the Rosaceae family. Selvaraj et al. (2015) also suggested nuclear ITS2 as an ideal barcode loci to identify the large plant family Apocynaceae with an accurate identification rate of 91% at the species level. Currently, researchers have broadened the application of the ITS2 region to the authentication of herbal materials. Founded on the numerous experiments and research, a publically available ITS2-based DNA barcoding system has been established to identify herbal materials (Chen et al., 2014). Zhao et al. (2015) indicated that ITS2 is an efficient tool to identify Acanthopanacis cortex and its adulterants. Moreover, Michel et al. (2016) found that ITS2 is a practical DNA barcode to authenticate herbal medicines sold in the New York City. Furthermore, Chen et al. (2017) developed a standardized barcode identification system for crude drugs in the Japanese pharmacopeia and again proved the identification capability of the ITS2 barcode. In the present study, we used the ITS2 barcode to discriminate medicinal species in *Cynanchum* and survey the authenticity of commonly used herbal materials in the medicine market to ensure their clinical safety and protect consumer interests.

## Materials and methods

### Plant materials

In this study, the capacity of the ITS2 barcode to identify medicinal species in *Cynanchum* was evaluated using 87 sequences representing 17 species of *Cynanchum*, from 33 vouchers collected in this study and 54 accessions downloaded from NCBI GenBank. All of the corresponding voucher samples were deposited in the Herbarium of the Institute of Medicinal Plant Development, Chinese Academy of Medical Sciences, Beijing, China. Moreover, 64 commercial crude drug samples from the three herbs (Cynanchi Paniculati Radix et Rhizoma, Cynanchi Stauntonii Rhizoma et Radix, and Cynanchi Atrati Radix et Rhizoma) recorded in the Chinese Pharmacopoeia were collected in herbal markets, hospitals, drug stores, and online shops from 12 provinces and municipalities in China to investigate their authenticity. Detailed information of the commercial samples is shown in **Table 2**. The crude drug samples were tested using a standard DNA barcoding database on the basis of the above 87 samples.

### DNA extraction, polymerase chain reaction (PCR) amplification, and sequencing

The surface of all herbal materials was cleaned with 75% ethanol to avoid fungal DNA contamination. About 60 mg of the materials were cut into pieces, added with 10% polyvinylpyrrolidone (PVP), and then ground with a FastPrep bead mill (Retsch MM400, Germany). The total DNA was extracted with a Plant Genome DNA Kit (Tiangen Biotech Co., China) in accordance with the manufacturer's instructions. The ITS2 sequences were amplified using universal primers ITS-S2F (5′-ATGCGATACTTGGTGTGAAT-3′) and ITS-S3R (5′-GACGCTTCTCCAGACTACAAT-3′) as previously described (Chen et al., 2010). Polymerase chain reaction (PCR) amplification was performed in a 25 μL reaction mixture containing 12.5 μL of 2 × PCR Master Mix (Aidlab Biotechnologies Co., Ltd.), 1.0 μL of each primer (2.5 μM), 2 μL (about 30 ng) of DNA templates, and filled with double-distilled water. The reactions were performed with the following thermal program: 94°C for 5 min and 40 cycles of 94°C for 30 s, 56°C for 30 s, and 72°C for 45 s, followed by 72°C for 10 min. The PCR products were sequenced by the Major Engineering laboratory of the Chinese Academy of Agricultural Sciences (Beijing, China).

### Data analysis

The original sequences of all 87 samples were assembled using CodonCode Aligner V5.2.0 (CodonCode Co., USA). The assembled sequences were annotated and trimmed to obtain the complete ITS2 region based on a hidden Markov model (Keller et al., 2009). All the ITS2 sequences obtained were aligned using MEGA 6.0. The genetic distances were calculated based on the Kimura 2-parameter (K2P) model using MEGA 6.0 (Tamura et al., 2013) to evaluate inter-specific and intra-specific variations. The average inter-specific distance, minimum inter-specific distance, and average theta prime were calculated to evaluate the inter-specific divergences using the K2P model. The average intra-specific distance, coalescent depth, and theta were used to represent the intra-specific variation based on the K2P model (Meyer and Paulay, 2005; Chen et al., 2010). BLAST1 and the nearest distance methods were both used to evaluate the species authentication efficacy (Ross et al., 2008). Neighbor-joining (NJ) tree and maximum-likelihood (ML) tree were constructed with MEGA 6.0 (Tamura et al., 2013) and performed with 1,000 bootstrap replicates. Moreover, Sequencher 5.0 software (Gene Codes Co., USA) was used to detect single-nucleotide polymorphisms (SNPs).

## Results

### Identification of medicinal species in *Cynanchum* using ITS2

#### Amplification, sequencing, and sequence characteristics

All of the materials collected were successfully amplified. The PCR products were all sequenced with high-quality bidirectional trace files. The ITS2 sequence obtained were submitted to GenBank under the following accession numbers: MF004207–MF004239 (see Table S1). The sequence lengths of the ITS2 region varied from 245 to 252 bp. The average GC content was 64.7%, ranging from 62.2 to 66.3%. The ITS2 length of the 17 medicinal species was 261 bp after alignment. A total of 50 interspecies variable sites were found in the ITS2 region. Detailed information about the ITS2 haplotypes from the 17 medicinal species in *Cynanchum* is provided in Figure S1.

#### Intra-specific and inter-specific genetic divergence analyses

Six metrics were calculated to characterize inter- vs. intra-specific variations based on 87 ITS2 sequences. As listed in Table 1, the inter-specific differences of *Cynanchum* species were greater than their intra-specific variations, and the ITS2 region had the potential to be used as a good DNA barcode for the identification of medicinal species in *Cynanchum*.

**Table 1:** Analysis of inter-specific divergence and intra-specific variation of 17 medicinal species in *Cynanchum*.

**Measurement**	**K2P-value**
Theta	0.0009 ± 0.0008
Coalescent depth	0.0012 ± 0.0025
All intraspecific distance	0 ± 0.0006
Theta prime	0.0457 ± 0.0176
Minimum interspecific distance	0.0165 ± 0.0164
All inter-specific distance	0.0395 ± 0.0271

#### Authentication efficiency analysis

**Figure 1:**
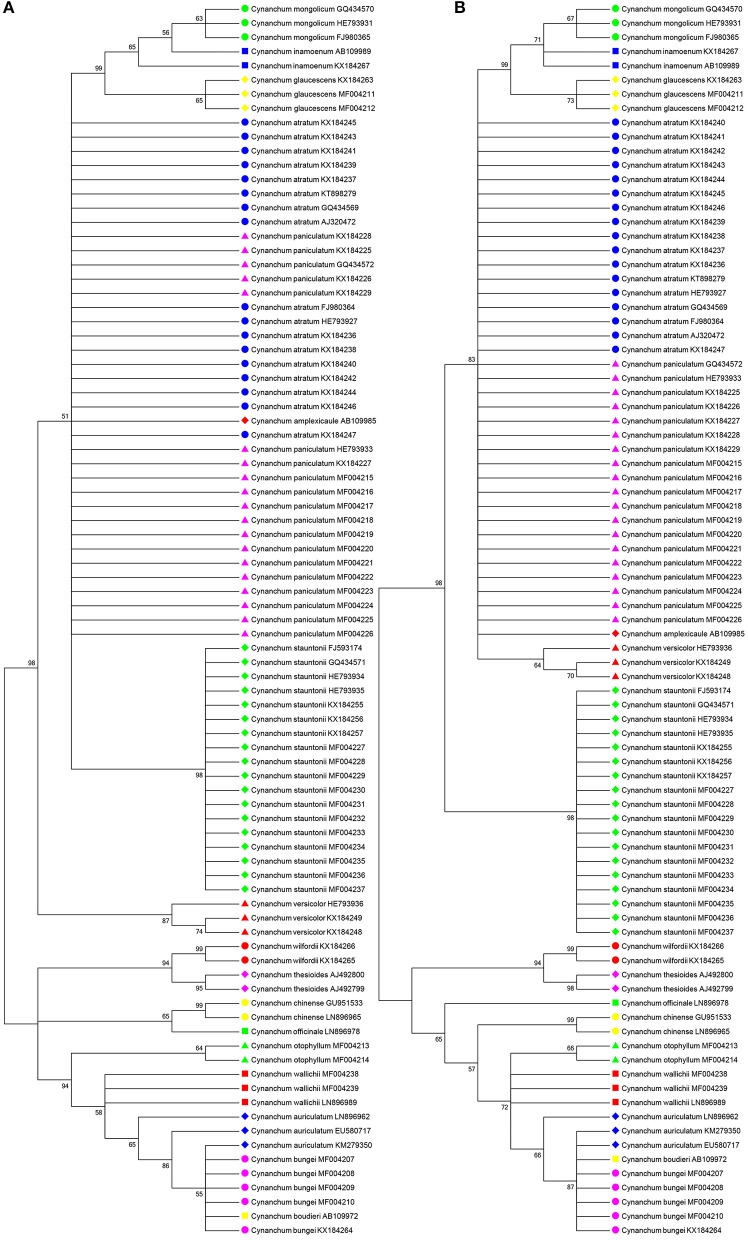
Neighbor-joining (NJ) tree and maximum-likelihood (ML) tree of *Cynanchum* species constructed with ITS2 sequences. The bootstrap scores (1,000 replicates) are shown (≥50%) for each branch. **(A)** refers to NJ tree; **(B)** refers to ML tree.

BLAST1 and the nearest distance methods were used to assess the authentication efficiency of ITS2 for *Cynanchum*, and the identification efficiency at the species level were 90.8 and 87.4%, respectively. Results showed the suitability of the ITS2 region to differentiate *Cynanchum* species. Meanwhile, NJ tree and ML tree were constructed to distinguish *Cynanchum* species based on all the 87 sequences of the ITS2 region. Consistent with the ML tree, the NJ tree showed that most species could be discriminated (Figure 1). Only a few species could not be identified, which included two pairs of species: *C. atratum, C. paniculatum*, and *Cynanchum amplexicaule*; *C. bungei, C. auriculatum*, and *C. boudieri*.

#### SNP analysis

SNP emerges when a single nucleotide differs in genome among members of a species, genus, or family; thus, it can be used to detect genetic diversity in plants, especially in relative species (Arif et al., 2010; Lee et al., 2012; Chen et al., 2013). For the two pairs of species that could not be discriminated using the NJ tree and ML tree, their ITS2 sequences were separately analyzed for SNPs at the inter-specific level. A stable C nucleotide deletion was found at the position of 52 bp between *C. paniculatum* and *C. atratum* on the basis of 36 ITS2 sequences, which could be used to distinguish the two species accurately (see Table S2). Simultaneously, no SNP was observed in the other pair of species (*C. bungei, C. auriculatum*, and *C. boudieri*).

### Survey of commercial drugs in the chinese pharmacopoeia

Of the 64 commercial crude drug samples from the three herbs recorded in the Chinese Pharmacopoeia, 49 were amplified and 44 were sequenced successfully. Among the 44 samples with high-quality bidirectional sequences, 11 were labeled as Cynanchi Atrati Radix et Rhizoma, 17 were labeled as Cynanchi Paniculati Radix et Rhizoma, and 16 were labeled as Cynanchi Stauntonii Rhizoma et Radix. Results showed that all the 11 Cynanchi Atrati Radix et Rhizoma samples were adulterants. Of these, five samples (WBJ01, WBJ02, WBJ03, WSX02, and WLN02) were authenticated as *C. glaucescens*, three samples (WYN01, WHN01, and WJL01) were authenticated as *Cynanchum mongolicum*, two samples (WAH01 and WSX01) were authenticated as *Ampelopsis japonica*, and the remaining one (WHB01) was authenticated as *Cynanchum inamoenum*. No adulterants were found in the 33 other samples from Cynanchi Paniculati Radix et Rhizoma and Cynanchi Stauntonii Rhizoma et Radix. Detailed information about the identification results of the test samples is provided in Table 2.

**Table 2:** Characteristics and identification results of the 64 commercial crude drug samples from the three herbs recorded in the Chinese Pharmacopoeia.

**Sample no**.	**Name of commercial crude drug samples**	**Best hit**	**Test results**	**Purchased place**
XCS01	Cynanchi Paniculati Radix et Rhizoma	*Cynanchum paniculatum*	G	Chengdu, Sichuan
XHB01	Cynanchi Paniculati Radix et Rhizoma	*Cynanchum paniculatum*	G	Anguo, Hebei
XHB02	Cynanchi Paniculati Radix et Rhizoma	*Cynanchum paniculatum*	G	Anguo, Hebei (Online)
XHB03	Cynanchi Paniculati Radix et Rhizoma	Unsuccessful amplification	/	Cangzhou, Hebei
XAH01	Cynanchi Paniculati Radix et Rhizoma	*Cynanchum paniculatum*	G	Bozhou, Anhui (Online)
XAH02	Cynanchi Paniculati Radix et Rhizoma	*Cynanchum paniculatum*	G	Bozhou, Anhui (Online)
XAH03	Cynanchi Paniculati Radix et Rhizoma	*Cynanchum paniculatum*	G	Bozhou, Anhui (Online)
XYN01	Cynanchi Paniculati Radix et Rhizoma	Unsuccessful sequencing	/	Yunnan
XYN02	Cynanchi Paniculati Radix et Rhizoma	*Cynanchum paniculatum*	G	Yunnan
XGX01	Cynanchi Paniculati Radix et Rhizoma	*Cynanchum paniculatum*	G	Yulin, Guangxi
XHN01	Cynanchi Paniculati Radix et Rhizoma	*Cynanchum paniculatum*	G	Zhengzhou, Henan
XHN02	Cynanchi Paniculati Radix et Rhizoma	*Cynanchum paniculatum*	G	Zhengzhou, Henan
XHN03	Cynanchi Paniculati Radix et Rhizoma	Unsuccessful amplification	/	Xinxiang, Henan
XGS01	Cynanchi Paniculati Radix et Rhizoma	*Cynanchum paniculatum*	G	Lanzhou, Gansu
XBJ01	Cynanchi Paniculati Radix et Rhizoma	*Cynanchum paniculatum*	G	Beijing
XBJ02	Cynanchi Paniculati Radix et Rhizoma	*Cynanchum paniculatum*	G	Beijing
XZJ01	Cynanchi Paniculati Radix et Rhizoma	*Cynanchum paniculatum*	G	Hangzhou, Zhejiang
XZJ02	Cynanchi Paniculati Radix et Rhizoma	*Cynanchum paniculatum*	G	Hangzhou, Zhejiang
XSX01	Cynanchi Paniculati Radix et Rhizoma	Unsuccessful amplification	/	Hanzhong, Shanxi
XSX02	Cynanchi Paniculati Radix et Rhizoma	Unsuccessful amplification	/	Shaanxi
XJL01	Cynanchi Paniculati Radix et Rhizoma	Unsuccessful sequencing	/	Jilin
XLN01	Cynanchi Paniculati Radix et Rhizoma	*Cynanchum paniculatum*	G	Fushun, Liaoning
XLN02	Cynanchi Paniculati Radix et Rhizoma	*Cynanchum paniculatum*	G	Fushun, Liaoning
QSC01	Cynanchi Stauntonii Rhizoma et Radix	Unsuccessful amplification	/	Yibin, Sichuan
QSC02	Cynanchi Stauntonii Rhizoma et Radix	*Cynanchum stauntonii*	G	Chengdu, Sichuan
QHB01	Cynanchi Stauntonii Rhizoma et Radix	*Cynanchum stauntonii*	G	Anguo, Hebei
QHB02	Cynanchi Stauntonii Rhizoma et Radix	*Cynanchum stauntonii*	G	Anguo, Hebei (Online)
QHB03	Cynanchi Stauntonii Rhizoma et Radix	Unsuccessful sequencing	/	Cangzhou, Hebei
QAH01	Cynanchi Stauntonii Rhizoma et Radix	*Cynanchum stauntonii*	G	Bozhou, Anhui (Online)
QAH02	Cynanchi Stauntonii Rhizoma et Radix	*Cynanchum stauntonii*	G	Bozhou, Anhui (Online)
QAH03	Cynanchi Stauntonii Rhizoma et Radix	*Cynanchum stauntonii*	G	Bozhou, Anhui (Online)
QYN01	Cynanchi Stauntonii Rhizoma et Radix	*Cynanchum stauntonii*	G	Yunnan (Online)
QYN02	Cynanchi Stauntonii Rhizoma et Radix	Unsuccessful sequencing	/	Yunnan
QGX01	Cynanchi Stauntonii Rhizoma et Radix	*Cynanchum stauntonii*	G	Yulin, Guangxi
QHN01	Cynanchi Stauntonii Rhizoma et Radix	*Cynanchum stauntonii*	G	Zhengzhou, Henan
QHN02	Cynanchi Stauntonii Rhizoma et Radix	Unsuccessful amplification	/	Zhengzhou, Henan
QHN03	Cynanchi Stauntonii Rhizoma et Radix	*Cynanchum stauntonii*	G	Xinxiang, Henan
QGS01	Cynanchi Stauntonii Rhizoma et Radix	Unsuccessful amplification	/	Lanzhou, Gansu
QBJ01	Cynanchi Stauntonii Rhizoma et Radix	*Cynanchum stauntonii*	G	Beijing
QBJ02	Cynanchi Stauntonii Rhizoma et Radix	*Cynanchum stauntonii*	G	Beijing
QBJ03	Cynanchi Stauntonii Rhizoma et Radix	*Cynanchum stauntonii*	G	Beijing
QQH01	Cynanchi Stauntonii Rhizoma et Radix	*Cynanchum stauntonii*	G	Qinghai
QSX01	Cynanchi Stauntonii Rhizoma et Radix	Unsuccessful amplification	/	Hanzhong, Shanxi
QSX02	Cynanchi Stauntonii Rhizoma et Radix	Unsuccessful amplification	/	Shaanxi
QJL01	Cynanchi Stauntonii Rhizoma et Radix	Unsuccessful amplification	/	Jilin
QJL02	Cynanchi Stauntonii Rhizoma et Radix	*Cynanchum stauntonii*	G	Jilin
QLN01	Cynanchi Stauntonii Rhizoma et Radix	Unsuccessful amplification	/	Fushun, Liaoning
QLN02	Cynanchi Stauntonii Rhizoma et Radix	*Cynanchum stauntonii*	G	Fushun, Liaoning
WSC01	Cynanchi Atrati Radix et Rhizoma	Unsuccessful amplification	/	Chengdu, Sichuan
WHB01	Cynanchi Atrati Radix et Rhizoma	*Cynanchum inamoenum*	A	Cangzhou, Hebei
WHB02	Cynanchi Atrati Radix et Rhizoma	Unsuccessful amplification	/	Anguo, Hebei
WAH01	Cynanchi Atrati Radix et Rhizoma	*Ampelopsis japonica*	A	Bozhou, Anhui (Online)
WAH02	Cynanchi Atrati Radix et Rhizoma	Unsuccessful sequencing	/	Bozhou, Anhui (Online)
WYN01	Cynanchi Atrati Radix et Rhizoma	*Cynanchum mongolicum*	A	Yunnan (Online)
WHN01	Cynanchi Atrati Radix et Rhizoma	*Cynanchum mongolicum*	A	Zhumadian, Henan
WBJ01	Cynanchi Atrati Radix et Rhizoma	*Cynanchum glaucescens*	A	Beijing
WBJ02	Cynanchi Atrati Radix et Rhizoma	*Cynanchum glaucescens*	A	Beijing
WBJ03	Cynanchi Atrati Radix et Rhizoma	*Cynanchum glaucescens*	A	Beijing
WQH01	Cynanchi Atrati Radix et Rhizoma	Unsuccessful amplification	/	Qinghai
WSX01	Cynanchi Atrati Radix et Rhizoma	*Ampelopsis japonica*	A	Hanzhong, Shanxi
WSX02	Cynanchi Atrati Radix et Rhizoma	*Cynanchum glaucescens*	A	Shaanxi
WJL01	Cynanchi Atrati Radix et Rhizoma	*Cynanchum mongolicum*	A	Jilin
WLN01	Cynanchi Atrati Radix et Rhizoma	Unsuccessful amplification	/	Fushun, Liaoning
WLN02	Cynanchi Atrati Radix et Rhizoma	*Cynanchum glaucescens*	A	Fushun, Liaoning

*G, genuine samples; A, adulterants; /, not applicable*.

## Discussion

### Difficulties in DNA extraction of herbal materials from *Cynanchum*

High-quality genomic DNA is the required precondition for DNA barcoding use. In general, extracting DNA from leaf-based medicines is comparatively easy, and only 10–20 mg of materials is needed in the experiment. Most herbs in *Cynanchum* are root- and rhizome-based and are rich in fiber and plant energy stores, thereby complicating DNA isolation (Chen et al., 2014). To solve this problem, researchers increased the amount of root- and rhizome-based herbs in their experiments to improve the success rate of extraction. For instance, Hou et al. (2016) increased the amount of materials to 40 mg and successfully obtained the DNA of Achyranthis Bidentatae Radix. Han et al. (2017) used 50 mg of herbal materials in DNA extraction. In our study, 40–50 mg of materials was initially used, but low-concentration and low-quality DNA was obtained. Subsequently, the utilization of 60 mg materials achieved the successful extraction of DNA. Aside from increasing the amount of material, several critical operation details remained in DNA extraction of herbal materials from *Cynanchum*. First, the materials were divided into two epoxy epoxide tubes (EP tube, 2.0 mL) before smashing, and about 30 mg of materials was contained in each tube to increase the cleavage of materials. Second, the extracts were concentrated into one spin column after freezing at −20°C for 20 min to concentrate the DNA. Third, 10% PVP-40 was used to remove polyphenols as the same as Hou's study for the sake of eliminating polysaccharides and ensuring DNA purity (Hou et al., 2016). Finally, heating DNA at 56°C overnight was performed to increase the cleavage of materials and the dissolution rate of DNA.

**Figure 2:**
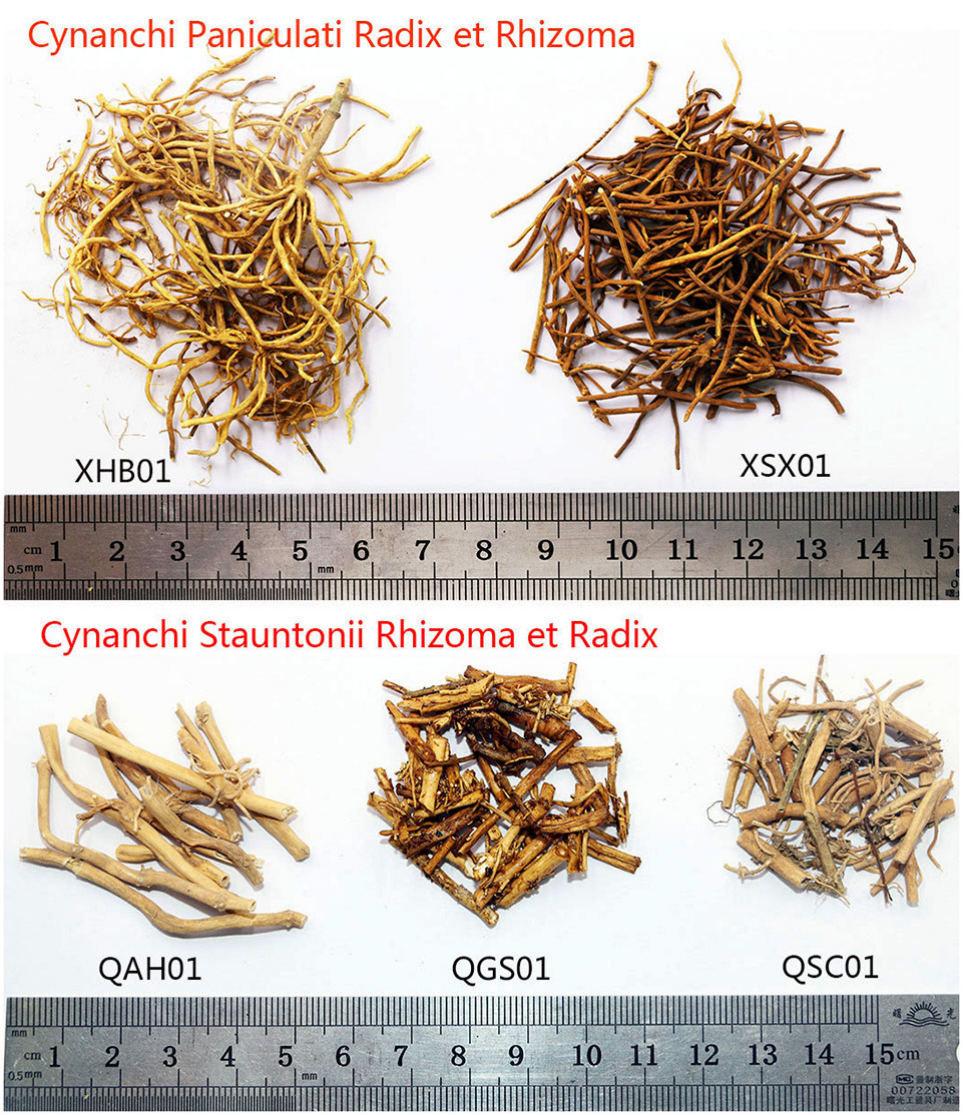
Some commercial crude drug samples of Cynanchi Paniculati Radix et Rhizoma and Cynanchi Stauntonii Rhizoma et Radix. XHB01 and QAH01 represent normal samples; XSX01 and QSC01 represent low-quality samples; QGS01 represents samples which were processed with honey.

Drug samples are stored for a long period before they are sold, along with the problem of DNA degradation. For 64 commercial crude drugs tested, the DNA of 49 samples was obtained successfully on the basis of the methods above, and no high-quality DNA was extracted from the 15 other samples even after numerous attempts. Among the 15 samples, samples XHN03, XSX01, XSX02, QSC01, and WQH01 were low in quality, probably kept for a long period, discolored, broken, and impure (Figure 2). Thus, DNA was highly degraded in the five samples, thereby inhibiting DNA extraction. In addition, samples QGS01, QSX01, QSX02, and QJL01 were all processed with honey, and high-temperature heat led to the heavy degradation of DNA and unsuccessful extraction of DNA (Figure 2). Furthermore, high-quality DNA was not obtained from some samples because they had sticky residues that could also inhibit the extraction of DNA even after washing three times with buffer.

### ITS2 barcode as an efficient tool to discriminate medicinal species in *Cynanchum*

The discrimination of authenticity of herbal medicines is the premise of clinical efficacy and safety. Traditional morphological identification relies on professional morphology knowledge and long-time experience. Close relatives with similar characteristics become much more difficult to identify correctly. This issue in *Cynanchum* is very apparent. Several species in this genus are closely similar in appearance, making them very hard to differentiate based on morphological identification. There are a total of 19 species used as herbs in *Cynanchum*, and almost all medicinal species (17 species) were collected and analyzed in the present research. The ITS2 barcode was used to discriminate these species and it was confirmed efficient in medicinal species identification in *Cynanchum*. First, significant differences were observed between the inter- and intra-specific divergences based on the 87 ITS2 sequences of *Cynanchum* species. Second, the ITS2 barcode had high discrimination capability using BLAST1 and the nearest distance methods with 90.8 and 87.4% identification efficiency at the species level. Third, our study indicated that not all medicinal species could be separated clearly based on ITS2 sequences because of the entirely same ITS2 region among some species, such as *C. bungei, C. auriculatum*, and *C. boudieri*. Therefore, other DNA regions might be required to achieve the complete identification of *Cynanchum* species. Finally, the SNP method was used to analyze the species that are difficult to be discriminated in this genus. At present, this method has been verified to be highly suitable for the analysis of related species. Chen et al. (2013) found that SNP-based molecular barcodes could accurately determine not only the *Panax* genus with closely related species but also the mixed powder of *Panax ginseng* and *Panax quinquefolius*. Zhao et al. (2015) showed that Acanthopanacis cortex and its closely related species could be identified through the three stable variations. Similarly, the stable variation site at 52 bp discovered between *C. paniculatum* and *C. atratum* in our study could also differentiate the two species efficiently. In general, ITS2 is a convenient and efficient barcode for medicinal species identification in *Cynanchum*, providing assistance for the discrimination of herbal materials and ensuring their safety use.

### DNA barcoding as a powerful technique supervising commercial herbal medicines from *Cynanchum*

**Figure 3:**
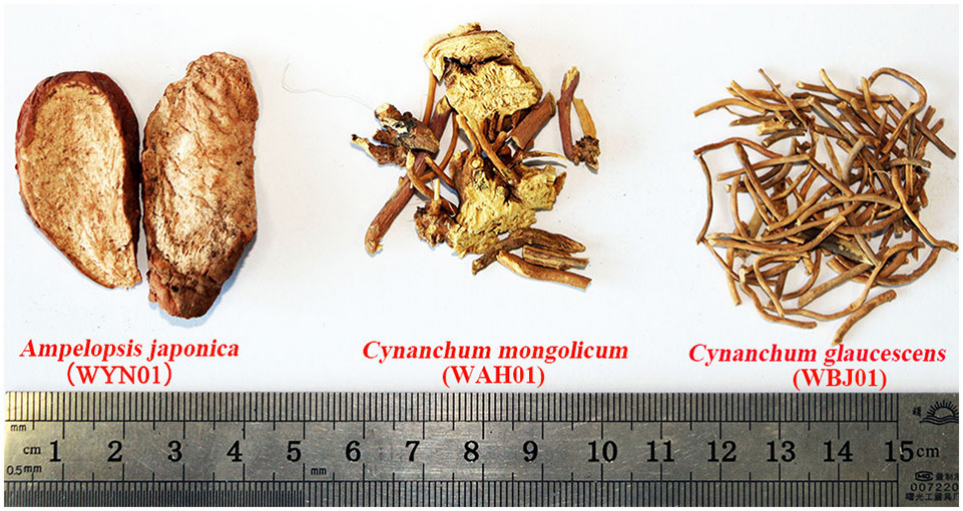
The main adulterants labeled as Cynanchi Atrati Radix et Rhizoma in Chinese medicine markets.

Recently, DNA barcoding has been applied to supervise the commercial products in the medicine market (Chen et al., 2015). For instance, Vassou et al. (2015) identified 25 raw drug samples of *Sida cordifolia* from 13 states in India, and their results demonstrated that none of the samples belonged to authentic species. Yu et al. (2016) collected 38 dried *Piper kadsura* samples from six medicine markets in China and found that 18.4% of the *P. kadsura* samples were not genuine. In our study, DNA barcoding was used to discriminate commercial herbal medicines from *Cynanchum*. The medicinal species in *Cynanchum* were widely distributed, but not all these herbal medicines are used frequently. Some herbal medicines, such as *C. inamoenum* and *C. chinense*, are only used locally and not well-known. Therefore, only the commonly used herbal medicines were investigated to ensure their clinical safety in our study. Fortunately, we found no adulterants in the test samples of Cynanchi Paniculati Radix et Rhizoma (Xuchangqing, 17 samples) and Cynanchi Stauntonii Rhizoma et Radix (Baiqian, 16 samples) from 12 provinces and municipalities. However, the potential safety problem of Cynanchi Atrati Radix et Rhizoma is especially prominent. All the 11 test samples were authenticated as adulterants. The three main species of the adulterants were *A. japonica, C. mongolicum*, and *C. glaucescens* (Figure 3). First, five samples were differentiated to be *C. glaucescens*, one of the original plants of Baiqian, showing that Baiqian and Baiwei are indeed used mistakenly because of their similar morphological characteristics and names. Second, two samples were identified as Ampleopsis Radix (Bailian). Bailian and Baiwei are completely different in appearance but similar in names, making them easily confused in the market. Third, three samples were authenticated as *C. mongolicum*, indicating that *C. mongolicum* is a common counterfeit drug of Baiwei. Moreover, *C. inamoenum* is another adulterant of Baiwei, which is uncommon in the medicine market. In sum, the adulterants of Baiwei have occupied the medicine market to a large extent at present, causing severe security risks of medicine use. Thus, effective measures must be taken to strengthen the supervision of Baiwei and ensure its accurate use.

At present, the online purchase of medicines has become easy with the rapid development of the Internet. Internet pharmacies are open all day, and people can compare the price and even procure some unavailable drugs in stores. Meanwhile, purchasing drugs online has become another way for patients to obtain low-quality or counterfeit drugs (Liang and Mackey, 2009; Orizio et al., 2011; Fittler et al., 2013). In our survey, the 64 test samples were not only bought online but collected from herbal markets, hospitals, or drug stores. Results indicated that counterfeit drugs existed in different selling channels. Two of the 19 commercial crude drug samples bought online in our study were confirmed as adulterants. Meanwhile, eight of the 45 samples collected from herbal markets, hospitals, or drug stores were authenticated as adulterants. Counterfeit drugs exist in nearly all selling channels, indicating that consumers, and patients are at risk of taking inappropriate medicines. Therefore, commercial products sold in the medicine market should be supervised to ensure their clinical safety and protect consumers' interests.

## Conclusion

In conclusion, ITS2-based DNA barcoding is a powerful tool to identify medicinal species in *Cynanchum* and to survey commonly used herbal medicines in the medicine market. Adulterants were found in the different selling channels, highlighting the necessity to supervise the medicine market. Given its numerous advantages, DNA barcoding is a potentially efficient tool for examining the origin of raw crude drugs and detecting adulterants accurately and effectively. Therefore, DNA barcoding should be applied in the supervision of commercial products in the medicine market. This technique could greatly improve the identification efficiency and accuracy of herbal medicines, thereby guaranteeing drug safety and protecting consumers' interests.

## Author contributions

XP conceived the study and participated in its design. MG and LR contributed samples and performed the experiments. MG and XP analyzed the data and drafted the manuscript. All authors have read and approved the final manuscript.

### Conflict of interest statement

The authors declare that the research was conducted in the absence of any commercial or financial relationships that could be construed as a potential conflict of interest.

## Supplementary material

Figure S1 Variable sites in the ITS2 sequence of the 17 *Cynanchum* species.

Table S1 Characteristics of the 17 medicinal species in *Cynanchum*.

Table S2 A stable SNP in ITS2 sequences from *C. paniculatum* and *C. atratum*.
