# Supplementary figures and images for: Inspecting the True Identity of Herbal Materials from Cynanchum Using ITS2 Barcode
Source: Front Plant Sci. 2017 Nov 13;8:1945. doi: 10.3389/fpls.2017.01945 (PMC5694171; doi:10.3389/fpls.2017.01945)

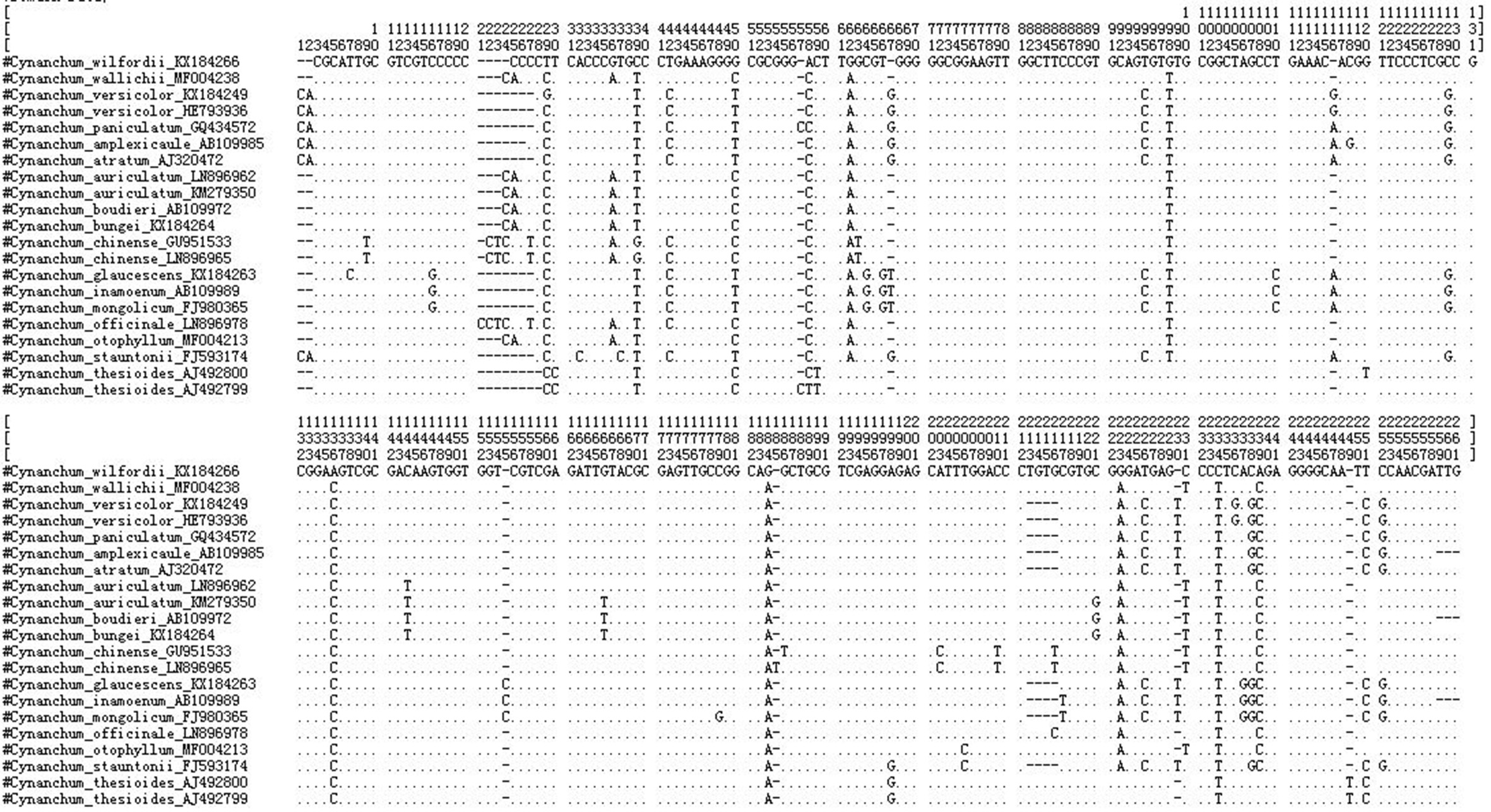

Supplement: Figure S1 — Variable sites in the ITS2 sequence of the 17 Cynanchum species. [file Image1.JPEG]
